# Conditional Deletion of ERK5 MAP Kinase in the Nervous System Impairs Pheromone Information Processing and Pheromone-Evoked Behaviors

**DOI:** 10.1371/journal.pone.0076901

**Published:** 2013-10-09

**Authors:** Junhui Zou, Daniel R. Storm, Zhengui Xia

**Affiliations:** 1 Toxicology Program in the Department of Environmental and Occupational Health Sciences, University of Washington, Seattle, Washington, United States of America; 2 Department of Pharmacology, University of Washington, Seattle, Washington, United States of America; University of Medicine & Dentistry of NJ - New Jersey Medical School, United States of America

## Abstract

ERK5 MAP kinase is highly expressed in the developing nervous system but absent in most regions of the adult brain. It has been implicated in regulating the development of the main olfactory bulb and in odor discrimination. However, whether it plays an essential role in pheromone-based behavior has not been established. Here we report that conditional deletion of the *Mapk7* gene which encodes ERK5 in mice in neural stem cells impairs several pheromone-mediated behaviors including aggression and mating in male mice. These deficits were not caused by a reduction in the level of testosterone, by physical immobility, by heightened fear or anxiety, or by depression. Using mouse urine as a natural pheromone-containing solution, we provide evidence that the behavior impairment was associated with defects in the detection of closely related pheromones as well as with changes in their innate preference for pheromones related to sexual and reproductive activities. We conclude that expression of ERK5 during development is critical for pheromone response and associated animal behavior in adult mice.

## Introduction

Vertebrates rely on chemosensory mechanisms to detect food sources, recognize social and mating partners, and avoid predators. It was originally thought that pheromones are primarily sensed through the vomeronasal organ (VNO) while odorants are detected through the main olfactory epithelium (MOE) [1–5]. Chemical signals acquired through the MOE and VNO are processed and integrated in the main olfactory bulb (MOB) and the accessory olfactory bulb (AOB), respectively. Detection of volatile odorants by the MOE is associated with several behaviors including food detection, whereas the detection of pheromones by the accessory olfactory system mediates several types of complex behavior including social and sexual activities. However, more recent studies have suggested that the MOE also plays a critical role in the detection of pheromones and the execution of gender-specific behavior such as male aggression and mating [6–9]. For example, male mice lacking functional CNGA2 (cyclic nucleotide-gated channel alpha 2) or AC3 (type-3 adenylyl cyclase), proteins that are required for the signal transduction through the MOE but are not expressed in the VNO, do not exhibit male dominance and male sexual activity [10,11]. Thus, both the MOE/MOB and VNO/AOB systems participate in pheromone detection and associated behaviors.

The extracellular signal-regulated kinase (ERK) 5 belongs to the mitogen-activated protein (MAP) kinase super family that includes ERK1/2, JNK, and p38 [12,13]. Although the N-terminal kinase domain of ERK5 shares a high degree of homology with ERK1/2, ERK5 signaling has unique regulatory properties and distinct biological functions that cannot be compensated by ERK1/2. Indeed, ERK5 null mice are embryonically lethal [14–16]. Our recent studies implicated ERK5 as a pro-neurogenic signaling molecule [17,18]. In addition, ERK5 plays a critical role in olfactory neurogenesis both during development and in adulthood [19–21]. Moreover, ERK5 is highly expressed in embryonic brain and conditional deletion of ERK5 in neural stem cells during development impairs neurogenesis of GABAergic interneurons in the MOB, resulting in fewer neurons in the MOB, a smaller MOB, and deficiencies in discrimination between structurally similar odorants [19]. In this study, we investigate if ERK5 function during development is important for pheromone detection and associated behaviors of adult animals.

## Materials and Methods

### Animals

The ERK5 deletion in neural stem cells was achieved as described by crossing an ERK5^loxP/loxP^ mouse line with a Nestin-Cre line [19]. Male Nestin-Cre/ERK5^loxP/+^ and female ERK5^loxP/loxP^ mice were mated to generate experimental Nestin-Cre/ERK5^loxP/loxP^ animals (ERK5 cKO), while ERK5^loxP/loxP^ and ERK5^loxP/+^ littermates were used as controls (ERK5 WT). Both ERK5 cKO and WT littermates were in mixed SV129 and C57BL/6 background. Animals were housed under standard conditions (12 h light/dark cycle) with food and water provided *ad libitum*. Three- to seven-month old sexually naive male mice were subjected to behavioral assays. Mice were individually housed and handled twice a day for 7 days prior to the assays. For all behavioral assays that were performed in the home cage, the bedding was last changed at least 4 days prior to each test to allow mice to establish their territories/home cage recognition and to avoid perturbation of the animals before behavior tests. Ovariectomized female and castrated male C57BL/6 mice, with surgeries performed at the age of 2-month old, were purchased from Charles River. They were used for urine collection and behavior experiments between the ages of 3 to 7-month old. All experimental procedures were approved and performed in accordance with the guidelines under the University of Washington Institutional Animal Care and Use Committee.

### Size measurement of AOB and quantification of NeuN^+^ cells in AOB

Animals of 28 days or 6 months of age were anesthetized and perfused transcardially with 4% PFA, post-fixed with 4% PFA overnight, and cryoprotected with 30% sucrose solution. Brains were then frozen in TFM^TM^ embedding medium and cut coronally at 14 μm in thickness. Sections were stained with cresyl violet or immonostained with anti-NeuN antibody as described [19]. Volumetric measurement of the AOB was conducted by using the Cavalieri Estimator probe of Stereo Investigator software (MBF Bioscience). And the quantification of NeuN^+^ cells in the AOB was conducted by using the Optical Fractionator probe of Stereo Investigator software. 

### Behavior assays

#### Intruder-resident assay

The intruder-resident assay was performed as described [10]. Briefly, a 3-month old sexually naive wild type SV129 male mouse was used as the intruder and introduced into the home cage of the subject male mouse for 15 min. Behavior was recorded with a video camera. Investigative and aggressive behaviors of the subject mouse were analyzed by an investigator blinded to the genotypes. Grooming and sniffing of the intruder by the subject mouse was defined as investigative behavior, while biting and wrestling initiated by the subject male mouse was defined as attacking behavior. Each intruder was used for 2 subject males per day, one in the morning and the other in the afternoon spaced by 4 h, for a total of two days. Intruders were returned to their home cages after each test. 

#### Reverse intruder-resident assay

The reverse intruder-resident assay was performed using 3- month old sexually naive wild type C57BL/6 male mice as the residents and the subject male mice as the intruders. Prior to the assay, the residents were singly housed with cages unchanged for 7 days. On the day of the test, a subject male mouse was placed into one corner of the home cage of the resident mouse for 15 min and the animals’ activity was recorded with a video camera. The subject male (intruder) was then returned to its home cage while the C57BL/6 resident male remained in the cage. Each resident mouse was used for 2 subject males per day, one in the morning and the other in the afternoon spaced by 4 h, for a total of two days. The aggressive behavior initiated by the subject male mouse was scored offline by an experimenter blind to the genotypes.

#### Male-female sexual behavior

The male-female sexual behavior assay was performed as described with modifications [10]. Ovariectomized C57BL/6 mice were used as female partners for this assay. The estrous status of female mice was achieved artificially by the injection of 20 μg estradiol benzoate (Sigma) at 48 and 24 h before the test and 500 mg progesterone (Sigma) at 4-7 h before the test [22,23]. At the time of the test, the estrous female was introduced into the home cage of the subject male mouse and the behavior of the subject male mouse was video recorded for 30 min. The duration of anogenital sniffing and mounting, and number of mounts by the subject male mice were scored by an investigator blinded to the genotypes and treatments. Each female mouse was used to test only one male subject per day, and two or three male subjects total during a period of 3 days. Female mice were returned to their home cages after the testing and housed in their home cages for at least 24 h before being used to test the next male subject. 

#### Mating choice assay

The mating choice assay was performed by introducing one male and one estrous female mouse simultaneously into the home cage of a subject test male mouse as described [24]. The estrous female was prepared as in the male-female sexual behavior assay. To avoid potential sexual interaction between the male and female targets, and the aggressive behavior of the male target toward the test subject male, castrated C57BL/6 males were used as the male targets because they have no sexual desire. However, these male targets were scented with normal male urine odor by swabbing urine from normal male mice on the back (50 μL) and genitourinary area (20 μL). The subject male was allowed to investigate both targets freely for 30 min and a video camera was used to record its behavior. The sexual behavior initiated by the subject test male mice toward the target male and the estrous female was scored from the video by an investigator blinded to the genotypes. Each target male or estrous female was used to test only one male subject per day and 2 or 3 total male subjects during a three-day period; they were returned to their home cages after each testing. The pairing of target male and estrous female was randomized. 

#### Urine habituation / discrimination assay

A male subject mouse was first presented for 1 min with a cotton swab laced with 20 µL water hanging from the top of the animal’s home cage with the cotton tips 8 cm above the cage floor. The presentation was repeated for a total of 4 times with 2 min inter-presentation intervals. Two minutes after the 4^th^ water presentation, a cotton swab laced with 20 µL undiluted urine samples was presented 1 min x 4 times in a similar manner, followed by presentations with additional urine samples. The duration of investigation for each presentation was scored. 

To collect male group A, male group B, and female urine for urine discrimination between different male groups and between male and female groups, 20 male and 10 female wild type SV129 mice, between 3- to 8-month old, were used for collection of urine samples. The twenty males were split randomly into two groups with 10 males per group and the 10 females constitute the third group. Urine was collected individually and pooled within each group with equal volume of urine from each individual. The pooled urine samples were labeled as male group A, male group B, and female urine. 

To collect female group A and female group B urine for urine discrimination between different female groups, 20 female wild type SV129 mice, between 3- to 8-month old, were used for collecting urine samples. Mice were split randomly into two groups (female group A and B) with 10 females per group. Urine was collected individually twice a day for 5 consecutive days and pooled within each group with equal volume of urine from each individual. 

Seven of 5-month old ovariectomized mice (Charles River, C57BL/6) were used for collecting urine from estrous and ovariectomized mice. Urine was collected individually twice a day for 14 consecutive days and pooled with equal volume of urine from each individual. This pooled and undiluted urine sample was designated ovariectomized urine. The ovariectomized mice were then injected intraperitoneally with 20 μg estradiol benzoate daily for seven consecutive days and 500 mg progesterone daily 24 h after each estradiol benzoate injection to achieve and maintain estrous status. Urine was collected individually daily between 4-7 h after the progesterone injection and pooled with equal volume of urine from each individual. This pooled urine was designated estrous urine. 

#### Urine preference assay

Pooled male or female urine samples were collected from 30-50 wild type SV129 mice at the age of 3-8 months. Ovariectomized and estrous urine samples were prepared as described above. Pooled and undiluted urine was used for this test. A subject male mouse was presented simultaneously with two cotton swabs laced with 20 µL urine collected from female vs. male, or ovariectomized vs. estrous females respectively, for 3 min. The placement of any specific urine sample to either left or right location was randomized among subject test mice. Duration of sniffing to each cotton swab was recorded. 

#### Open field assay

The open field assay was performed as described[25] 

#### Elevated plus maze assay

A beige elevated plus maze apparatus was used for this experiment (San Diego Instruments Co). The maze consists of two perpendicularly located open arms and closed arms with a center area. Each arm measures 50X5 cm and the center area measures 5X5 cm. The one-third distal portion of the two open arms and the two closed arms were defined as the open ends and the closed ends, respectively. The maze was placed in the center of a room with its stage 40 cm above the floor level and all the arms at least 50 cm away from any object in the room. Animals were placed in the center of the maze facing toward one of the open arms and allowed to freely explore the stage for 5 min. A video camera was installed onto the ceiling of the room and directly above the center of the maze. The video camera was connected with a computer and ANY-maze software (San Diego Instruments Co) was used to track and analyze the movement in a real-time mode. 

#### Light/dark box assay

The test was conducted in a 2-chamber shuttle box with an opaque divider in the middle of the box (Coulbourn Instruments). Each chamber measures 17X17X33 cm in dimensions. An opening measuring 6X6 cm was located at the center bottom of the divider. The walls of one chamber were made of black plexiglass while those of the other chamber were transparent. The ceiling of the box was made of a sheet of aluminum with a round hole measuring 1 cm in diameter over the center of the light chamber. Mice were habituated in the room for 1 h before the test with the room lights off. During the test, the room was not illuminated but the light compartment was lit by a 60W bulb closely placed over the hole on the ceiling. During the test, a mouse was first placed into the dark chamber and allowed to freely travel between the chambers for 5 min. The time spent in the light chamber was scored.

#### Forced swim test

The test was performed as described [26]. Briefly, a 4-L glass beaker (16.5 cm in diameter) filled with water (23-25 °C) to a depth of 17.8 cm was used as the apparatus. Mice were placed in the beaker and allowed to swim undisturbed for 6 min and then removed, dried and returned to their home cages. Water was changed between each subject. The entire session was recorded from the side by a video camera. The video was scored later by an experimenter blind to the genotypes for the latency to first episode of immobility, the duration of immobility and immobile episodes during the last 4 min of the session. 

#### Innate fear avoidance assay

The assay was performed in a three-chamber shuttle box made of clear plexiglass. Each chamber measured 21X19X12.5 cm in dimension and the chambers were divided by a piece of clear plexiglass with a semicircular opening (6 cm in diameter) at the center bottom. Before the test, an empty plastic chemical weighing boat measuring 4X4 cm was placed in each end chamber of the box. A mouse was then placed in the middle chamber of the box and allowed to freely investigate the box and weighing boats for 15 min. The mouse was then transferred to his home cage briefly, during which time the weighing boats were loaded with a piece of square filter paper (8X8 mm) soaked either with 50 µL DMSO or 1 mM 2,3,5-Trimethyl-3-thiazoline (TMT, Phero Tech) dissolved in DMSO, respectively. The same mouse was placed back into the middle chamber and allowed to investigate the box and weighing boats freely for 5 min. The time spent in each chamber was scored. 

### Testosterone measurement

Blood was collected from ERK5 cKO and wild type control mice. The serum testosterone level was measured by using an ACE competitive enzyme immunoassay kit (Cayman Chemical, Ann Arbor, MI) according to manufacturer’s instructions. 

### Statistical analysis

All data were expressed as mean ± standard error of the means (SEM). Comparison between the WT and ERK5 cKO groups was analyzed by Student’s t-test, two-tailed analysis. n.s., not significant; *, p<0.05; **, p<0.01; ***, p<0.001. n=9-14 for ERK5 WT group and 8-9 for ERK5 cKO mice in each test.

## Results

### Mice with conditional deletion of ERK5 in neural stem cells have smaller AOB

**Figure 1:**
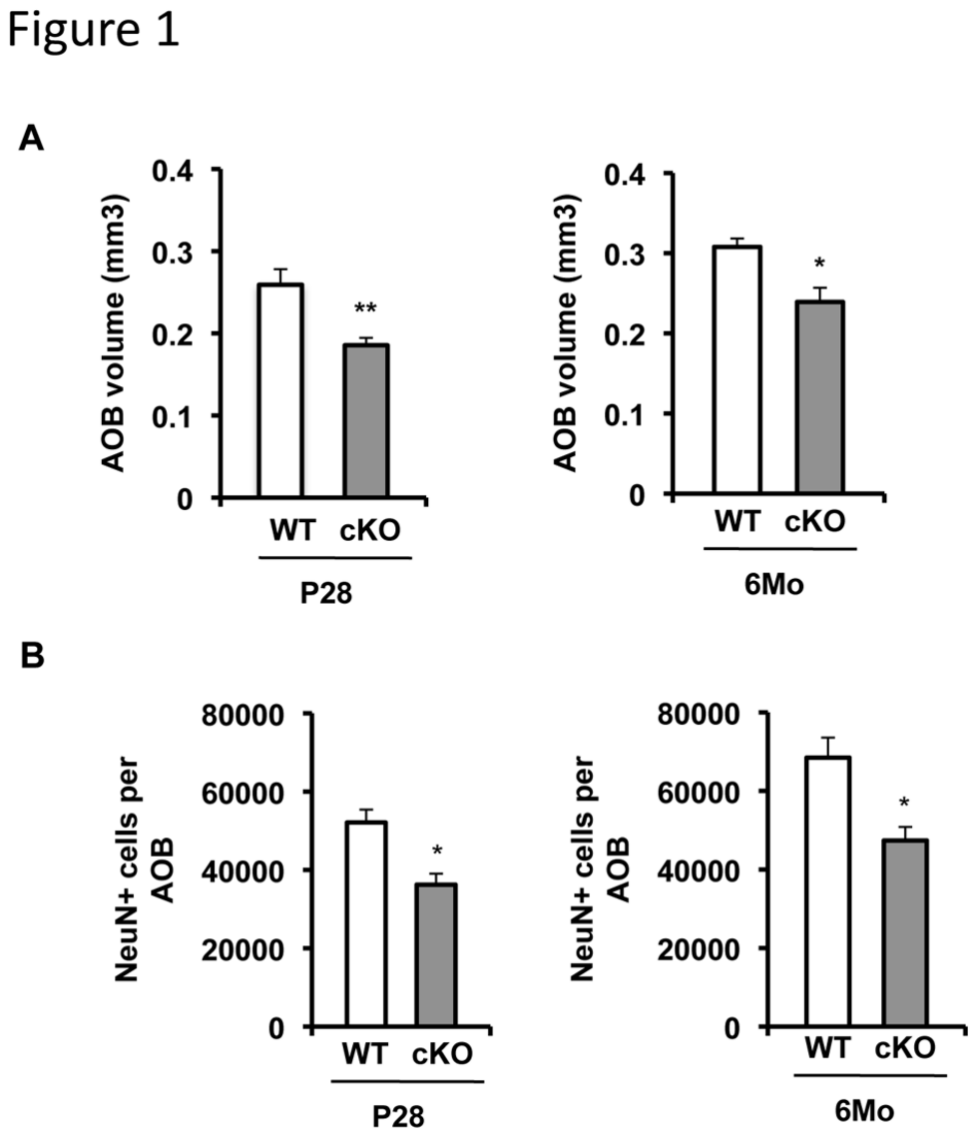
ERK5 cKO mice have smaller accessory olfactory bulb (AOB). ***A***, ERK5 deletion results in a smaller AOB at P28 and 6 months of age. ***B***, There are fewer NeuN^+^ neurons in the AOB of ERK5 cKO mice at P28 and 6 months. n=5 per genotype. *, p<0.05; **, p<0.01.

We previously reported that conditional deletion of ERK5 in neural stem cells (ERK5 cKO) abolishes MOB neurogenesis [19]. Since pheromone can be detected by both MOB and AOB, we examined if ERK5 deletion also affects the development of AOB. The relative AOB volume, measured using an unbiased stereological quantification technique, was 28% or 22% smaller in ERK5 cKO mice at postnatal day (P) 28 and 6 months (Figure 1A), respectively, compared with their littermate controls. To determine if the reduction in AOB size was due to reduced number of neurons generated, an antibody against NeuN, a marker for neurons, was used to label mature neurons in the AOB. Indeed, the total number of NeuN^+^ cells was reduced by 30% in ERK5 cKO mice, at both ages (Figure 1B). These data suggest that, in addition to MOB development, ERK5 function is also required for proper development of the AOB.

### Mice with conditional deletion of ERK5 in the nervous system do not discriminate between different groups of male or female urine

**Figure 2:**
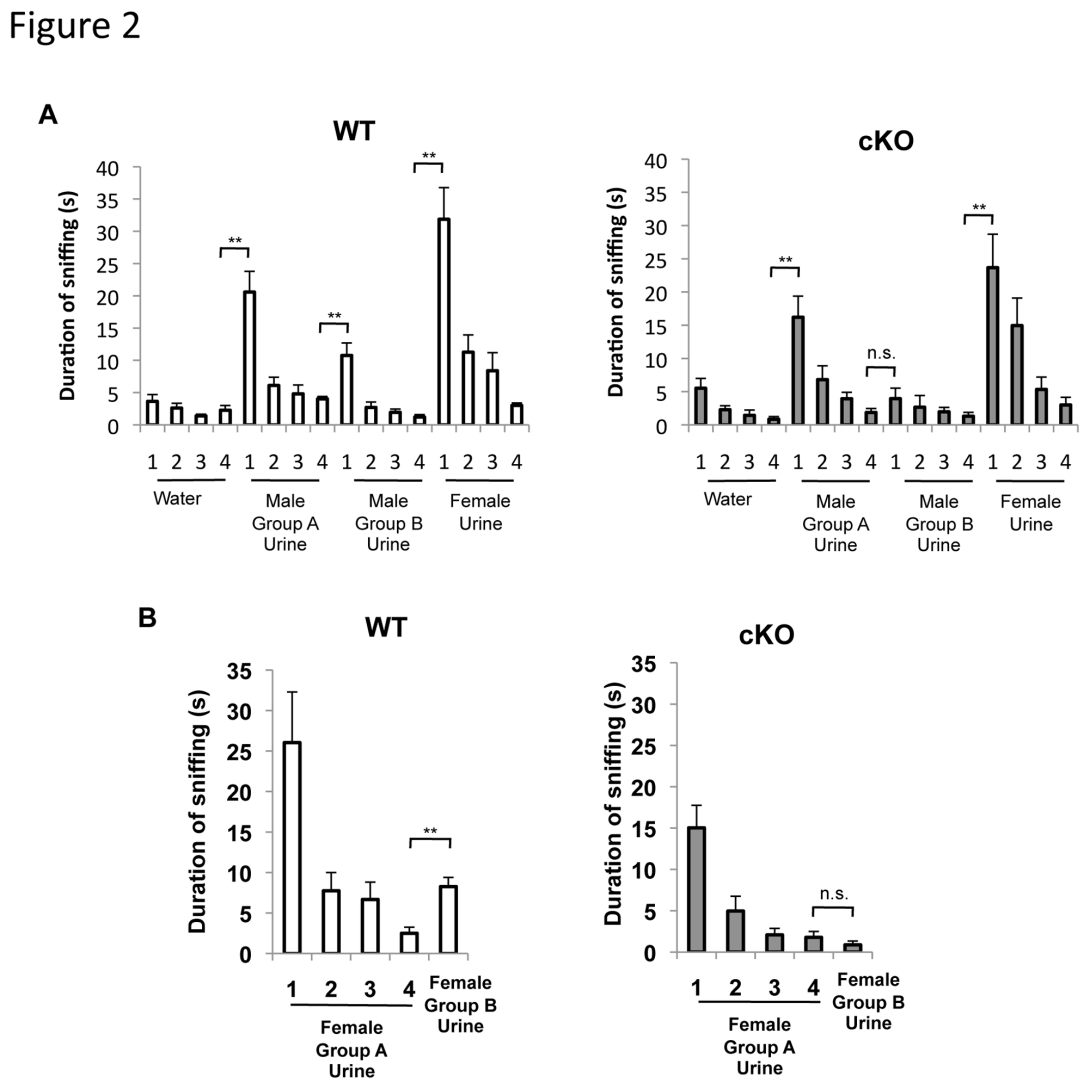
ERK5 cKO mice are deficient in discriminating urine samples from different groups of mice of the same gender. ***A***, ERK5 cKO mice are able to sense and discriminate between male and female urine but cannot discriminate urine samples from different groups of male mice in a standard habituation/dishabituation olfaction test. Naïve, adult mice were pre-trained with four presentations of water-soaked cotton swabs, then exposed sequentially to three urine samples collected from two groups of 10 male mice (Male Group A and B) and one group of 10 female mice. ***B***, Similarly, ERK5 cKO mice cannot distinguish between urine samples from Group A and B female mice. n.s., not significant; **, p<0.01.

 Conditional deletion of ERK5 in neural stem cells of ERK5 cKO mice impairs discrimination between pairs of structurally similar odorants [19]. To determine whether the ERK5 cKO mice are also deficient in pheromone detection, we subjected male ERK5 cKO and their wild type (WT) littermates to an olfactory habituation/discrimination assay using urine samples collected from male or female mice. Each test mouse was first habituated to the presence of a cotton swab laced with water in its home cage. This pre-training ensured that sniffing of subsequent urine presentations was not a response to the cotton swab as a novel object. Then, we presented the mouse with a cotton swab laced with urine collected and pooled from a group of ten male mice (male group A urine) for 4 trials. Both the WT and ERK5 cKO mice showed significantly increased duration of sniffing upon the first exposure of the urine sample compared to the 4^th^ presentation of the water-laced cotton swab, indicating that the animals detected the group A male urine sample (Figure 2A). Sniffing declined in subsequent trials because the olfactory information was no longer novel. 

 This test was repeated with urine samples pooled from a group of 10 different male mice (male group B urine) or 10 female mice (female urine) sequentially. The group A and group B male mice were split randomly from a cohort of 20 mice housed under similar conditions. The WT mice differentiated the subtle differences between male urine samples from group A and group B, and spent more time sniffing the first presentation of male group B urine relative to the 4^th^ presentation of male group A urine (Figure 2A). Interestingly, unlike their WT littermates, ERK5 cKO mice did not show increased sniffing towards group B male urine. However, this was not because ERK5 cKO mice generally lost interest in the urine-laced cotton swabs. Like their WT littermates, they showed interest in female urine when it was presented after the group B male urine. Similarly, WT littermates, but not ERK5 cKO mice, were able to differentiate urine samples collected from group A versus group B female mice and showed increased sniffing when female group B urine was presented after female group A urine samples (Figure 2B). These data suggest that although ERK5 cKO mice can clearly distinguish between pheromone-containing male and female urine, they cannot detect the subtle differences in the urine of different groups of mice within the same gender.

### ERK5 cKO male mice are impaired in their innate preference for normal female and estrous female pheromones

**Figure 3:**
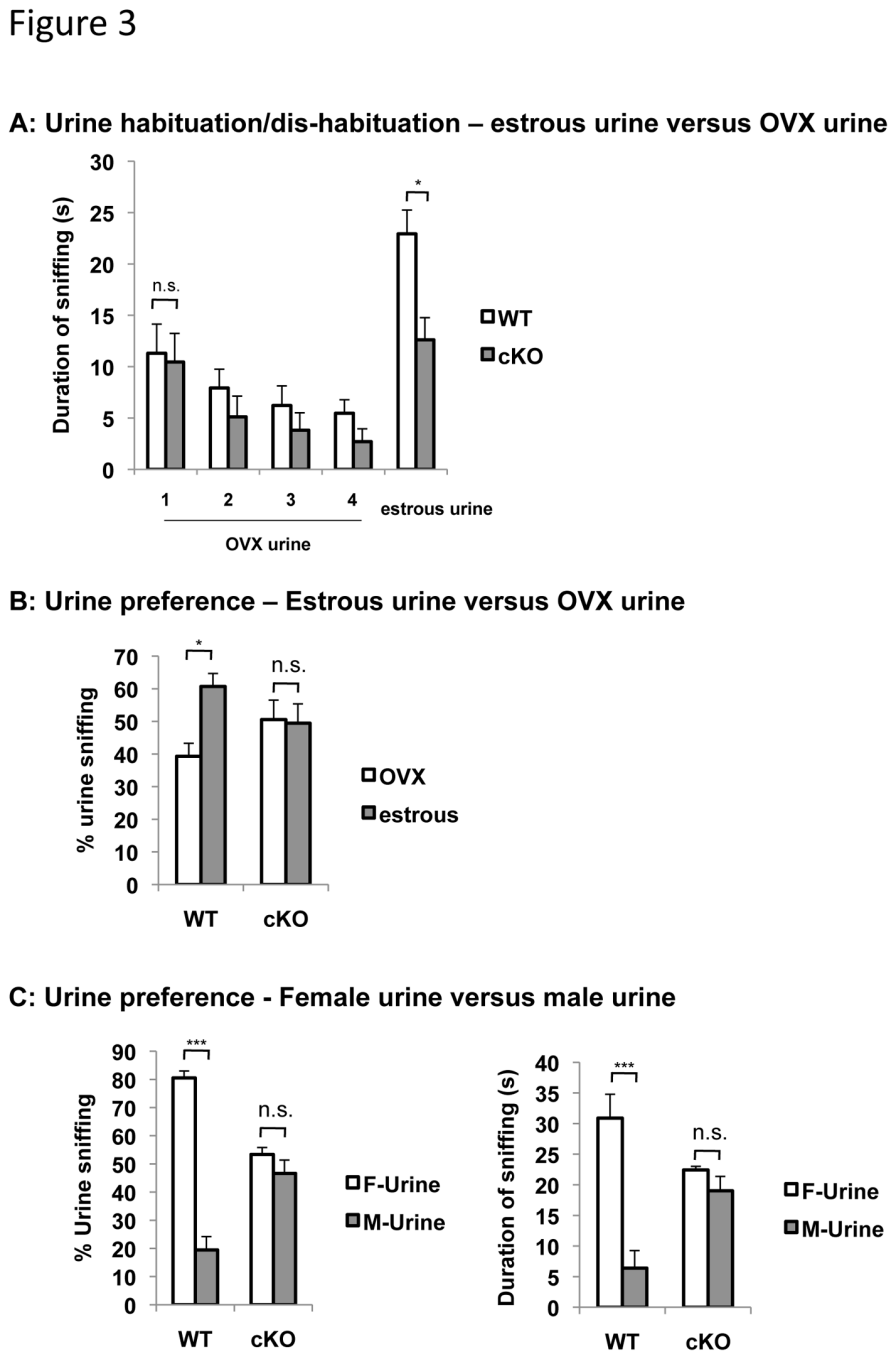
Conditional deletion of ERK5 impairs male mice’s preference for normal and estrous female pheromones. ***A***, ERK5 cKO mice are able to discriminate between the ovariectomized (OVX) urine and the estrous urine, although they display reduced level of interest to the estrous urine compared with their WT littermates. Ovariectomized mice were artificially cycled with the estradiol and progesterone and the urine collected before hormone injection was used as the ovariectomized urine, while urine collected after hormone injection was used as the estrous urine. The subject mice were presented with one cotton swab laced with ovariectomized urine for 4 trials followed by one trial of the estrous urine. ***B***, Male ERK5 cKO mice do not prefer urine from estrous females over that from ovariectomized females. Mice were presented simultaneously with two cotton swabs laced with urine samples collected and pooled from a group of ovariectomized female mice either before or after the artificial cycling with the estradiol and progesterone. ***C***, Male ERK5 cKO mice lost their innate preference for the female urine versus male urine. Mice were presented simultaneously with two cotton swabs laced with male or female urine samples respectively. n.s., not significant; *, p<0.05; ***, p<0.001.

 Mice were also tested for their detection of urine samples from female mice in different reproductive stages, estrous and non-estrous. To avoid potential confounding from volatile odor and pheromone components that are unrelated to the female reproductive stages, a cohort of ovariectomized female mice were artificially cycled with estradiol and progesterone. The urine collected before hormone injection was used as non-estrous urine, designated ovariectomized (OVX) urine, and that collected after hormone injection was used as estrous urine. The differences in the two urine samples are likely largely due to different pheromones related to the reproductive stages of female mice since the mice were maintained under identical conditions before and after hormone injection. We presented male mice with four trials of ovariectomized urine followed by one trial of estrous urine. The WT and ERK5 cKO mice were equally interested in the ovariectomized urine at its first presentation, and their sniffing declined in subsequent three sequential exposures to the same urine sample (Figure 3A). When the estrous urine was presented as the 5^th^ presentation, both mice showed increased sniffing compared to the 4^th^ presentation of the ovariectomized urine, suggesting that *ERK5* deletion does not affect the animals’ ability to discriminate the estrous urine from the ovariectomized urine. However, ERK5 cKO mice sniffed the estrous urine significantly less than WT mice. This suggests that although male ERK5 cKO mice are able to sense the differences between the ovariectomized and estrous urine samples, they were not as interested in the estrous female pheromones as their WT littermates. 

 The preference of the male mice for urine samples of ovariectomized or estrous female mice was also examined. Male mice were presented simultaneously with cotton swabs laced with ovariectomized or estrous urine, and their sniffing duration toward each sample was scored. WT mice spent 60% of the time sniffing estrous urine versus 40% of the time sniffing ovariectomized urine (Figure 3B). This preference for urine from the estrous mouse was not observed with ERK5 cKO mice; they spent equal amounts of time investigating both urine samples. 

 When examined for their preference for male versus female urine, WT male mice demonstrated a strong preference for female urine; they spent 80% of the time investigating female urine versus 20% of the time investigating male urine (Figure 3C). In contrast, ERK5 cKO male displayed no preference for either urine samples. These data suggest that ERK5 deletion abolishes male mice’s preference for female pheromones over male pheromones, and for female pheromones present in the reproductive stage (estrous urine) over those in the non-reproductive stage (ovariectomized female urine).

### ERK5 cKO male mice show reduced sexual activity

**Figure 4:**
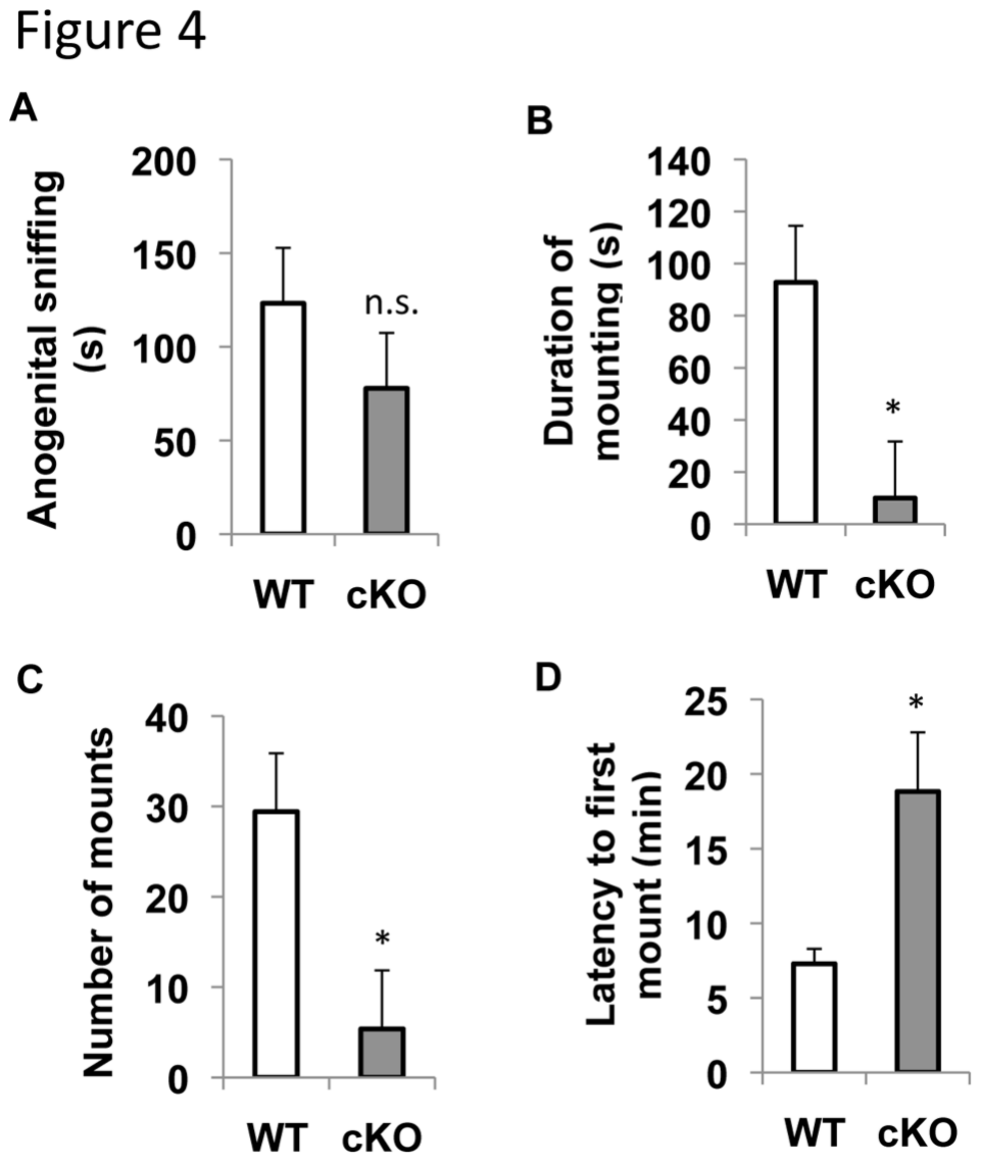
Male ERK cKO mice show reduced mating activity. The mating behavior assay was performed by introducing an estrous female mouse into the home cage of a subject male mouse. The estrous status of the female mouse was achieved by injecting estradiol and progesterone into an ovariectomized female mouse. Both investigative (sniffing) and sexual (mounting) behaviors were quantified. ***A***, Time spent in anogenital sniffing. ***B***, Duration of mounting. ***C***, Number of mounts. ***D***, Latency to the first mount. n.s., not significant; *, p<0.05.

 Because the ability to detect and distinguish pheromones is essential for mating and courtship behavior in mice, we investigated whether the impaired pheromone response of ERK5 cKO mice leads to changes in mating behavior. Male ERK5 cKO mice and their WT littermates were examined for mating behavior by introducing an estrous female into their home cages for 30 min. Ovariectomized female mice were artificially cycled with estradiol and progesterone and used as estrous females to ensure their receptiveness towards the test male’s sexual behavior. As expected, WT mice were highly motivated in anogenital investigation of the females and mounted the females (Figure 4). Although ERK5 cKO male mice also displayed anogenital investigation toward the females, both the number of mounts and duration of mountings were greatly reduced compared to their WT littermates (Figure 4A-C). Furthermore, the latency to first mounting increased from 7 min in WT to 18 min in cKO (Figure 4D). These data demonstrate that ERK5 deletion impairs sexual behavior of male mice. 

**Figure 5:**
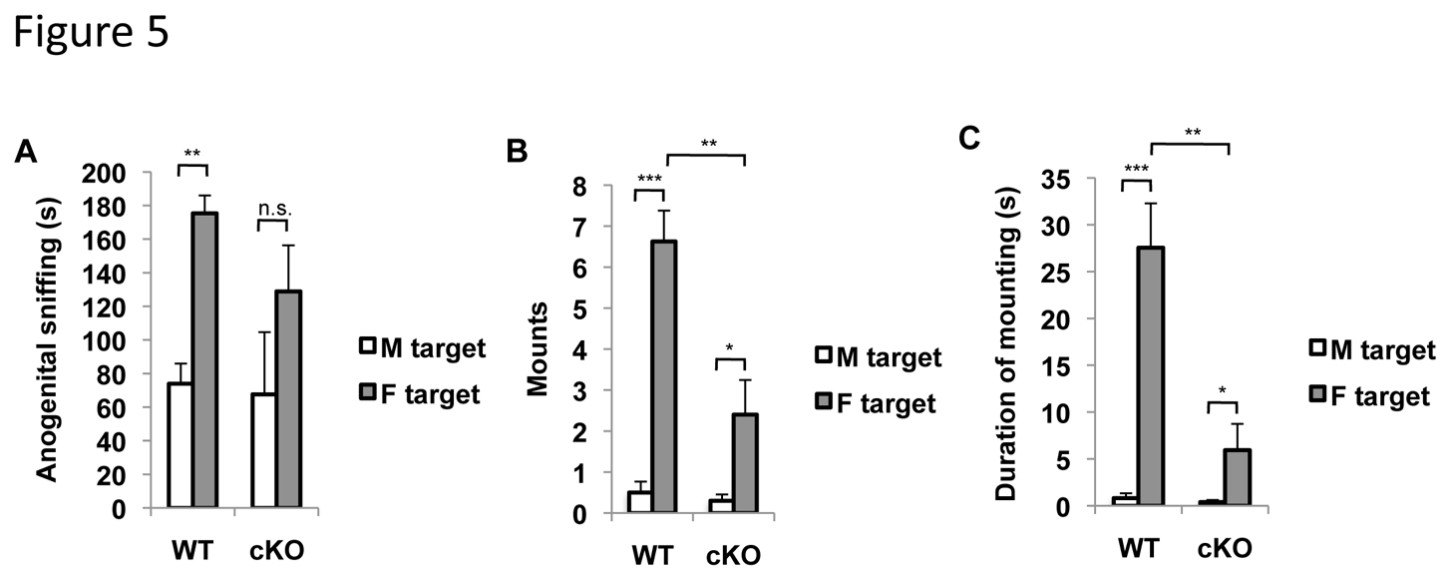
ERK5 deletion does not alter the mating choice of male mice. The mating choice assay was performed by introducing an estrous female and a male target mouse simultaneously into the home cage of the test male mouse. An ovariectomized female mouse was injected with estradiol and progesterone to achieve estrous status and used as the female target. The male target was a castrated male mouse odorized with the urine from normal male mice. Both investigative (sniffing) and sexual (mounting) behaviors were quantified. ***A***, Time spent in anogenital sniffing. ***B***, Number of mounts. ***C***, Duration of mounting. n.s., not significant; *, p<0.05; **, p<0.01; ***, p<0.001.

 Because ERK5 cKO mice showed no preference for female urine over male urine, we examined if the reduced mating towards females is because ERK5 cKO mice have altered sexual orientation and mating preference. To address this issue, we performed a mating choice assay by introducing two target mice simultaneously, one male and one estrous female, into the home cage of a test subject male ERK5 cKO or WT mouse. Again, estrous females were those ovariectomized female mice artificially cycled with estradiol and progesterone to ensure their receptiveness towards the sexual behavior of the male test subject. To avoid aggressive behavior toward the test subject male or mating behavior towards the female, castrated male mice were used as the target males. These mice were swabbed with urine from other sexually mature male mice to “scent” them with male pheromones. Both WT and ERK5 cKO male mice performed substantial anogenital sniffing toward the two target mice (Figure 5A). Although ERK5 cKO mice conducted fewer mounts and shorter total mounting in general than their WT littermates, they still mated only with female mice but not with male mice (Figure 5B,C). These data indicate that male ERK5 cKO mice show reduced mating activity but no change in sexual orientation or mating preference. 

### ERK5 cKO mice are deficient in male aggression

**Figure 6:**
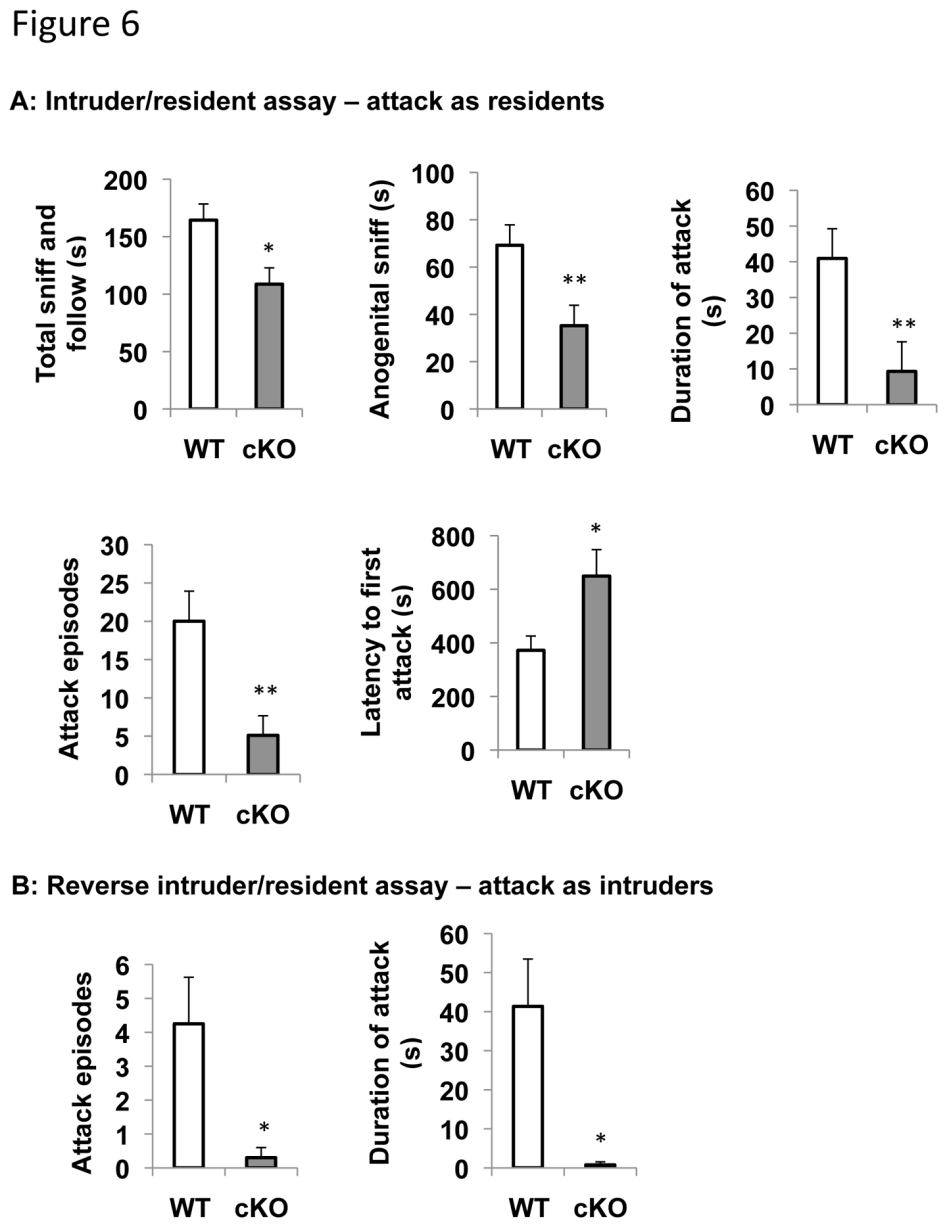
ERK cKO male mice are deficient in male-male aggression. ***A***, A standard intruder-resident assay was conducted by introducing a sexually naïve normal SV129 male mouse into the home cage of a test male mouse. Both investigative (total sniffing and following, anogenital sniffing) and aggressive behaviors (duration of attack, attack episodes, latency to first attack) of the test male mice were quantified. ***B***, A reverse intruder-resident assay was conducted by introducing a test male mouse into the home cage of a sexually naïve normal C57BL/6 male mouse. The aggressive behavior (duration of attack, attack episodes) initiated by the male ERK5 cKO or WT mice toward the resident mice was quantified. *, p<0.05; **, p<0.01.

 In addition to mating, pheromone detection is also critical for other gender-specific behavior including male-male aggression. To determine whether ERK5 deletion affects conspecific aggression of male mice, we conducted an intruder-resident assay. In the first set of the assays, a sexually mature but inexperienced SV129 male mouse was used as the intruder and introduced into the home cage of a male ERK5 cKO or WT mouse (resident). The investigative and aggressive behavior of the resident ERK5 cKO or WT mouse towards the intruder was scored. The investigative behavior, measured by total sniffing, following, and anogenital sniffing, was reduced in ERK5 cKO male mice compared to WT littermates (Figure 6A). In contrast to WT littermates, ERK5 cKO mice initiated very few attacks, measured by the duration of attack and the number of attack episodes. Furthermore, the latency to first attack was much longer with ERK5 cKO mice. 

 A reverse intruder-resident assay was also performed wherein a test subject ERK5 cKO or WT male mouse was introduced into the home cage of a sexually naïve normal C57BL/6 male mouse. C57BL/6 is a less aggressive mouse strain compared to the SV129. The C57BL/6 residents initiated minimum aggression toward either WT or ERK5 cKO intruder males in this experiment, allowing the aggression behavior initiated by the test subject male (the intruder) been scored. Unlike their WT littermates, male ERK5 cKO mice rarely initiated attacks against the resident males (Figure 6B). These data demonstrate that ERK5 cKO mice are impaired in male-male aggression.

### TMT does not elicit fear response in ERK5 cKO mice

**Figure 7:**
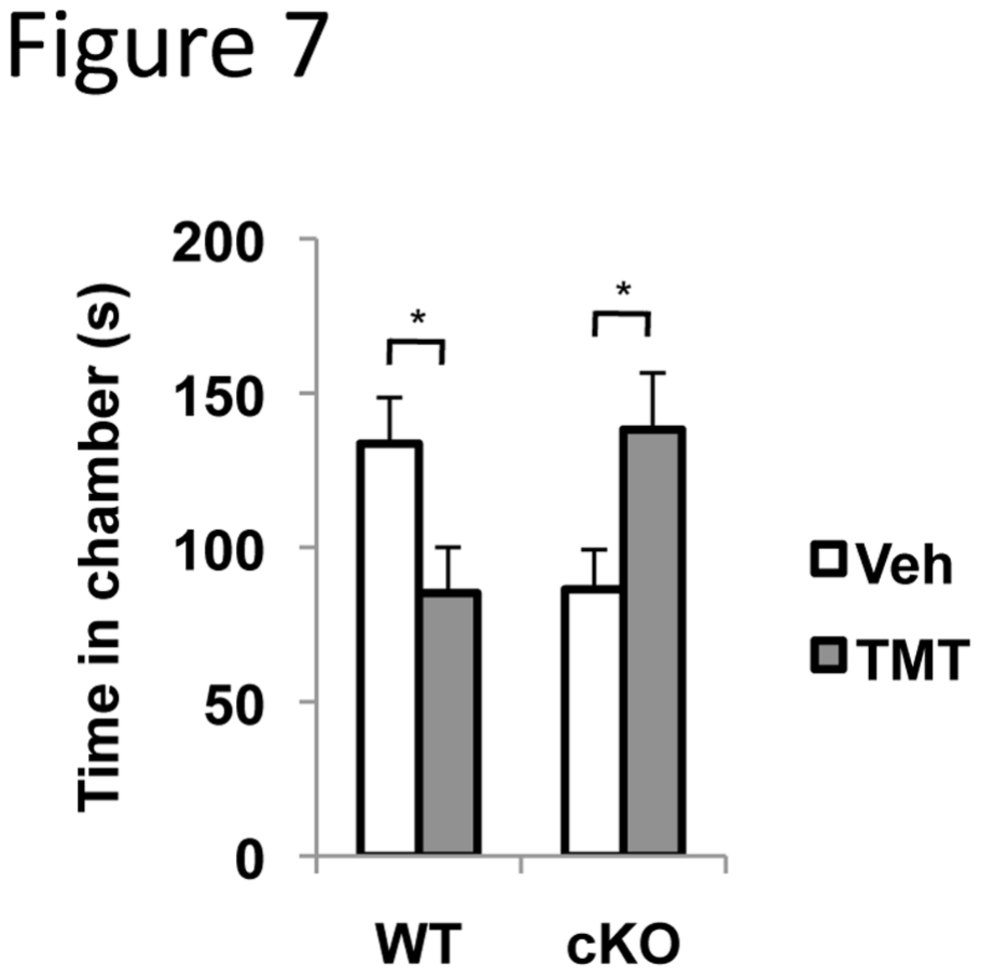
ERK5 cKO male mice do not show innate-fear avoidance towards TMT. Male ERK5 cKO mice and their WT littermates were introduced into the middle chamber of a three-chamber shuttle box with vehicle (Veh) or 1 mM TMT, a predator odor, placed in the either of two end chambers separately. Time spent in each chamber was quantified. *, p<0.05.

 To evaluate the impact of ERK5 deletion on response to a fear-eliciting predator odor, we performed an innate fear avoidance assay using a 3-chamber apparatus with TMT. TMT is secreted by the anal gland of fox, and is known to elicit innate fear and alarm in rodents [27,28]. Male ERK5 cKO or their WT littermates were introduced into the middle chamber of a 3-chamber shuttle box and TMT or vehicle control was placed in either of the two side chambers, respectively. The duration of investigation of ERK5 cKO or WT mice toward each chamber was quantified. When normal mice detect TMT, they typically avoid the chamber as a manifestation of fear. Indeed, WT mice spent less time investigating the chamber with TMT than the chamber with vehicle-control (Figure 7). In contrast, ERK5 cKO mice visited the TMT chamber even more than the vehicle-control chamber, indicating that they were able to detect TMT as an odor but they failed to elicit an associated fear response. These data also suggest that the impaired mating and male aggression behavior of ERK5 cKO mice is not due to heightened fear in general.

### ERK5 deletion does not alter mobility, activity, increase anxiety, nor cause depression

**Figure 8:**
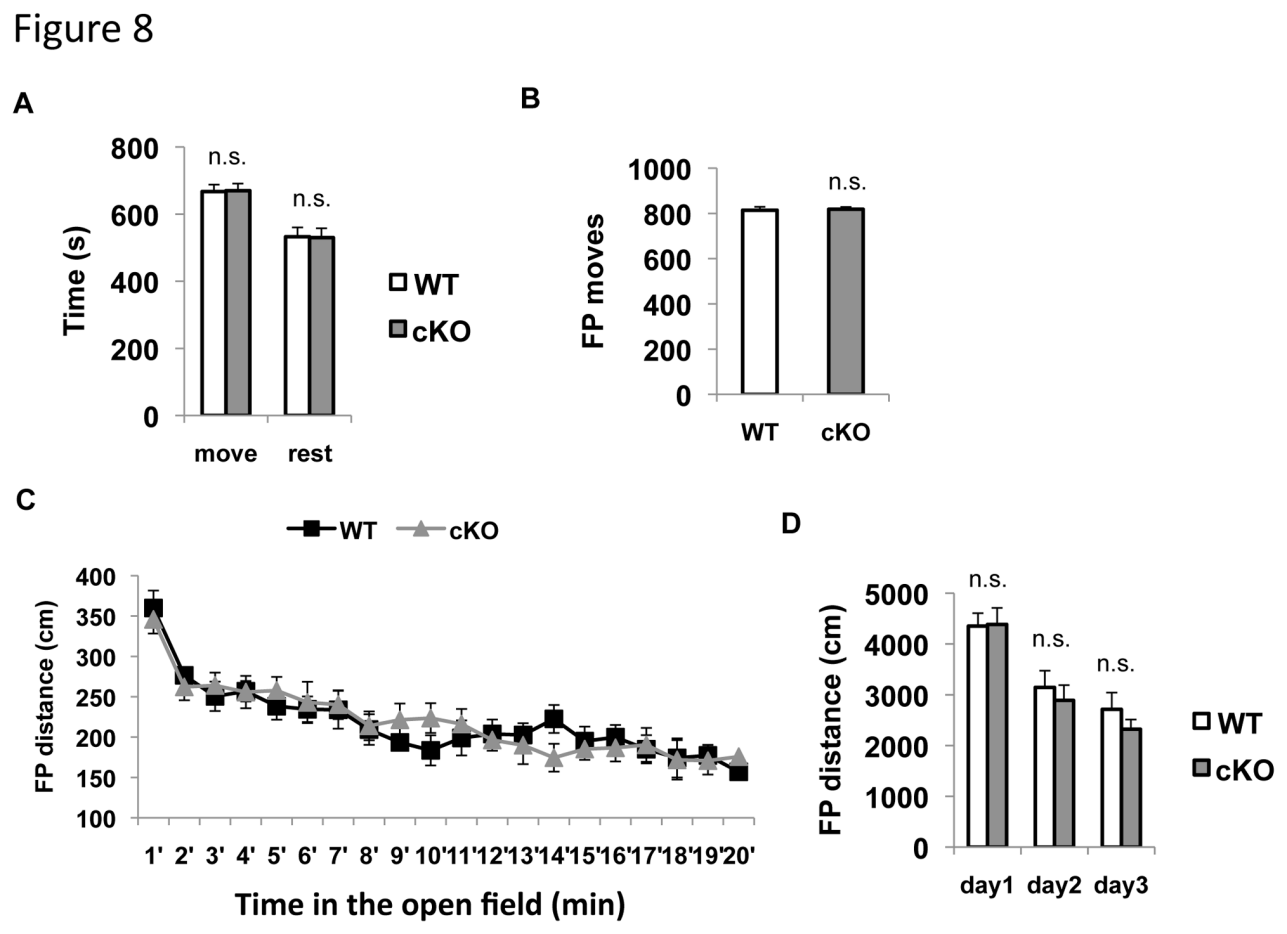
ERK5 deletion does not alter the levels of mobility and activity in an open field test. Mice were subjected to an open field assay on three consecutive days with a 20-min trial on each day. ***A***, Total time spent in moving or resting in the first day of the test. ***B***, Total number of floor plane (FP) moves during the first day of the open field training. ***C***. ERK5 deletion does not affect the habituation of the mice to the open field area on the first day of the training. The 20-min trial of the first day training was computationally split into 20 segments with 1 min per segment and the FP moving distance in each segment was measured. Both ERK5 cKO and WT mice moved actively in the first segment and the movement declined as they habituate to the open field. ***D***, Both ERK5 cKO and WT male mice habituated similarly to the open field arena during repeated exposure in 3 consecutive days. Their activity was quantified by the FP distance traveled on each day. n.s., not significant.

 Others factor that could affect the mating and aggression behavior of ERK5 cKO mice including mobility, activity, anxiety, depression, and the level of testosterone were examined. There was no reduction in serum levels of testosterone, a major male hormone, in ERK5 cKO male mice (data not shown). In an open field assay, both male ERK cKO and their WT littermates spent the same amount of time moving in the arena and traveled equal distance when first exposed to the arena (Figure 8A,B). Also, both mice habituated to the arena with declining activity during continued (Figure 8C) and repeated exposures for 3 days (Figure 8D). This data suggests that ERK5 deletion does not alter the levels of mobility and activity of male mice. 

**Figure 9:**
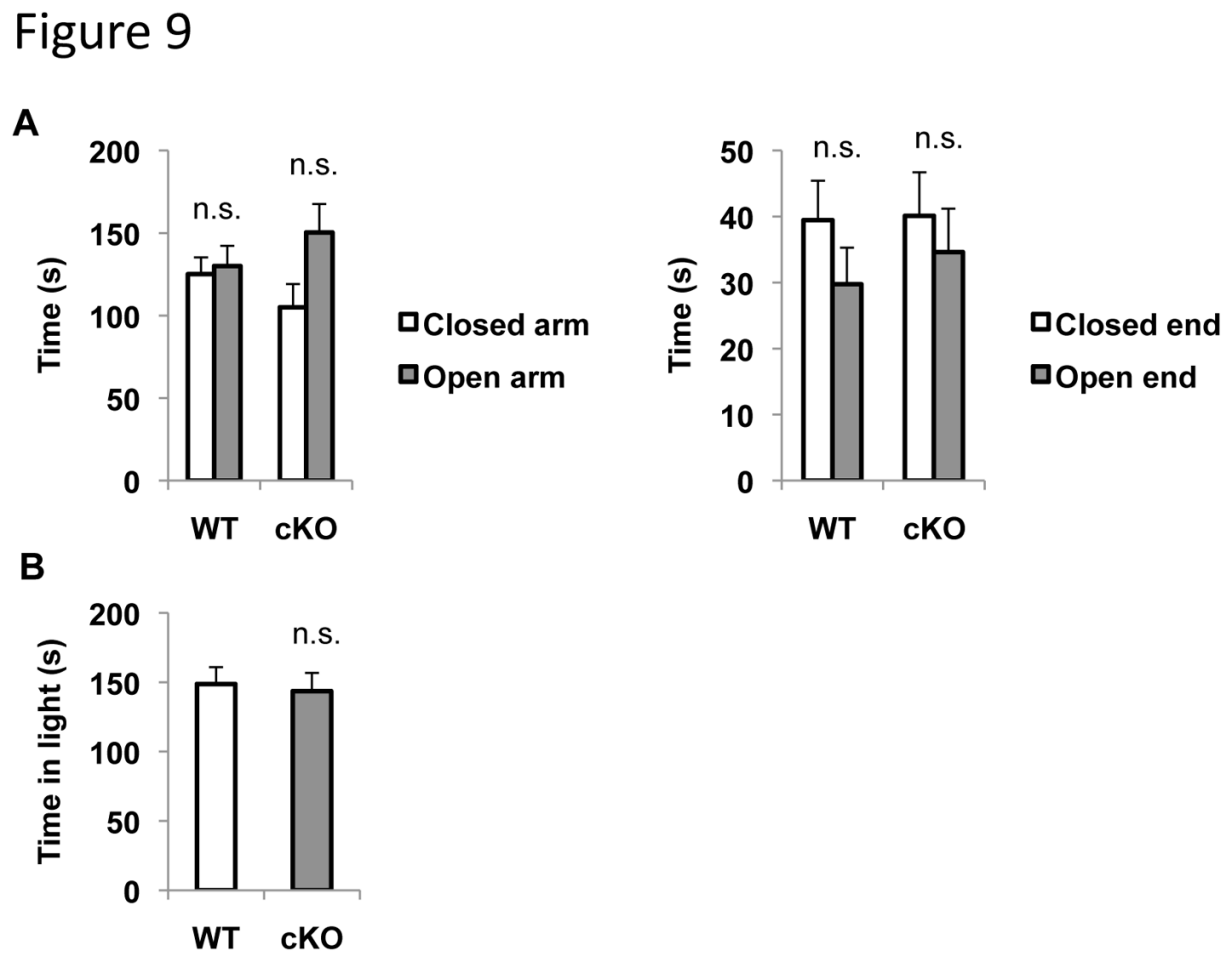
ERK5 deletion does not increase anxiety. ***A***, ERK5 cKO mice do not show a preference for the closed arms or ends in the elevated plus maze assay, a characteristic feature of elevated anxiety. The distal one-third of the open and closed arms were defined as the open and closed ends respectively. ***B***, ERK5 cKO and WT male mice spent an equal amount of time investigating in the light chamber of the dark/light box s., not significant.

Mice were also subjected to the elevated plus maze and dark/light box assays, two commonly used behavioral tests for anxiety. Reduced time spent in the open arms or open ends in the elevated plus maze, or decreased time spent in the light chamber of the dark/light box assay signals anxiety. In the elevated plus maze assay, ERK5 cKO mice spent a similar amount of time investigating the open arms as well as the open ends compared to their WT littermates (Figure 9A). Similarly, in the dark/light box assay, ERK5 cKO mice spent the same amount of time in the light chamber as their WT littermates (Figure 9B). These data suggest that ERK5 deletion does not increase anxiety in these animals.

**Figure 10:**
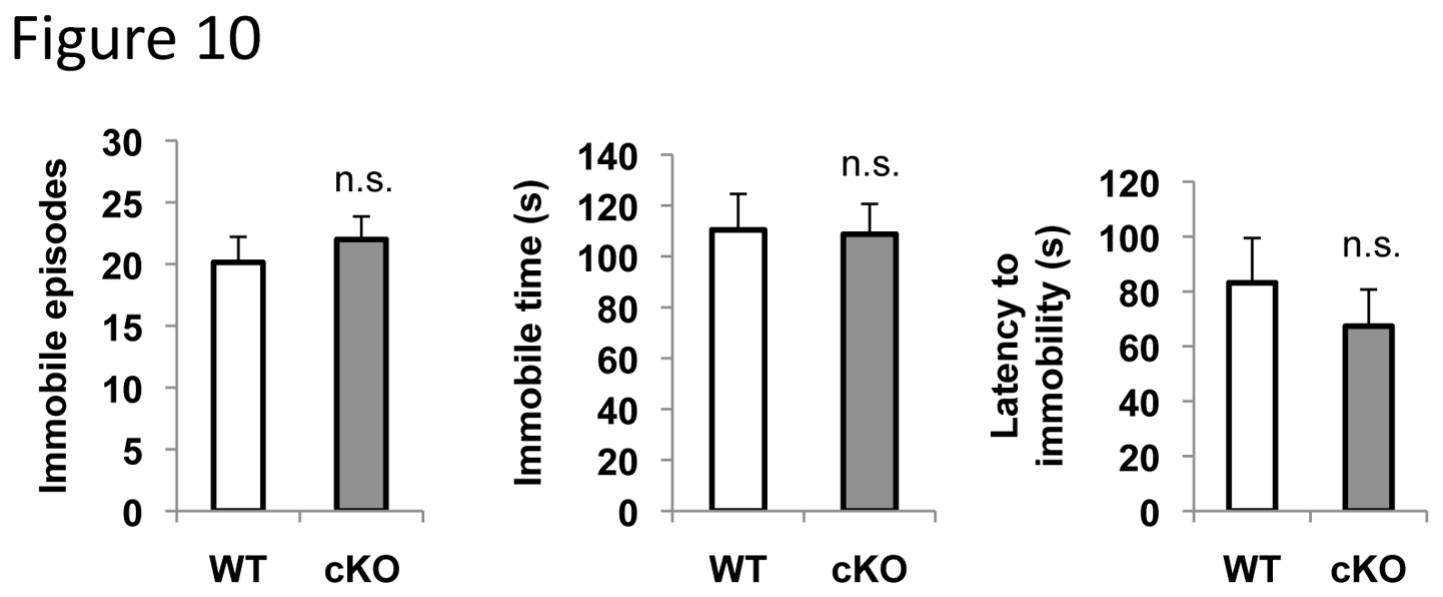
ERK5 deletion does not cause depression in a forced swim test. Mice were subjected to a 6-min session of a forced swim test; the duration of total immobile time and total immobile episodes during the 2-6 min timeframe of the session, as well as the latency to first immobile episode were scored. n.s., not significant.

The animals were evaluated in a forced swim test, a standard and widely used method to measure depression in mice. ERK5 cKO mice had similar immobile episodes, total immobile time, and latency to first immobile episode as their WT littermates (Figure 10), demonstrating that they are not more immobile nor do they display early onset of despair. These data suggest that ERK5 deletion does not cause depression.

## Discussion

 Although the ERK5 MAP kinase is highly expressed in the developing nervous system [17,18], several studies reported that it is dispensable for the development of the brain [29,30]. We also observed that conditional deletion of ERK5 in neural stem cells does not cause gross abnormalities of the brain [19]. However, we recently reported that the MOB of ERK5 cKO mice is smaller due to reduced number of GABAergic interneurons generated during development [19]. ERK5 cKO mice are also deficient in odor discrimination between structurally similar odorants. We report here that ERK5 cKO mice also have smaller AOB. The objective of this study was to investigate whether the smaller MOB and AOB in ERK5 cKO mice lead to changes in pheromone-mediated behaviors.

 Male ERK5 cKO mice are capable of detecting and differentiating distinct pheromone-containing urine samples, such as those from male versus female mice, or from ovariectomized versus estrous female mice, when presented sequentially [23,31]. However, unlike their WT littermates, ERK5 cKO mice cannot distinguish between closely related urine samples, such as those collected from two groups of male mice or from two cohorts of female mice, mice that are otherwise of the same gender, similar age and housed under identical conditions. This suggests that ERK5 cKO mice may be deficient in detecting subtle differences of pheromones present in closely related urine samples, much like their deficiency in discriminating between structurally similar odorants [19].

 Normal male wild type mice show strong preference for female urine over male urine, and for urine from estrous female mice over that from ovariectomized females when the two urine samples are presented simultaneously [11,23]. However, male ERK5 cKO mice completely lost such innate preference and spent equal time investigating both samples. Because the ovariectomized and estrous female urine samples were collected from the same cohort of ovariectomized female mice before and after hormone injection, the differences in these two urine samples are likely largely due to changes of pheromones after hormone injection. These data suggest that although ERK5 cKO mice can detect distinct pheromones present in the urine, they may be deficient in the processing and interpretation of the perceived pheromone information. Interestingly, although ERK5 cKO mice were able to detect TMT, a predator odor that generally elicits fear response in rodents, they did not respond by avoiding TMT as their WT littermates did. Rather, they spent more time investigating TMT. This finding further supports the hypothesis that conditional ERK5 deletion in the nervous system impairs the ability to interpret chemical and pheromone information appropriately.

 Male ERK5 cKO mice exhibit impaired pheromone-based behavior. For example, they show reduced mating towards female mice and less male-male aggression behavior, either as intruder or as resident. Besides chemosensory cues, physical mobility, activity, mood status, and the level of male hormone may also influence male-male aggression and mating [24,32,33]. We demonstrated that ERK5 deletion does not decrease activity, mobility, or circulating male hormone testosterone. It also does not cause anxiety or depression, nor enhance fear. Thus, aberrant detection and interpretation of pheromone information likely play a major role in deficient male aggression and mating behavior of ERK5 cKO mice.

Detection of pheromones is thought to be critical for gender-specific behavior in mice, including male-male aggression and mating behavior [34–36]. Interestingly, male ERK5 cKO mice can sense and detect pheromone-containing urine samples, and distinguish between female versus male urine, and ovariectomized versus estrous urine almost as well as WT mice, yet they show very little mating activity or male aggression either as the intruder or the resident. Although male ERK5 cKO mice showed equal interest in female urine and male urine, they still only mated with females but not with males whenever they mated. Thus, the ability to detect pheromones *per se* may be necessary but not sufficient to initiate sexual activity or male aggression. The inability of ERK5 cKO mice to properly process and interpret pheromone signals may contribute significantly to their impaired behavior.

In summary, our data suggest an important function of ERK5 during development of the nervous system in the regulation of pheromone information processing and in associated pheromone-evoked behavior in male mice.
